# Tolerability and safety of Octagam^®^ (IVIG): a post-authorization safety analysis of four non-interventional phase IV trials 

**DOI:** 10.5414/CP202782

**Published:** 2016-10-10

**Authors:** Wolfgang Frenzel, Stefan Wietek, Tor-Einar Svae, Anette Debes, Daniel Svorc

**Affiliations:** 1 Octapharma Pharmazeutika Produktionsges.m.b.H., Vienna, Austria,; 2 Octapharma USA Inc., Hoboken, NJ, USA, and; 3 Octapharma GmbH, Langenfeld, Germany*; *At the time of study realization.

**Keywords:** immunodeficiency, IVIG, PASS, safety, tolerability

## Abstract

Objective: To evaluate the tolerability and safety of Octagam^®^ 5% and 10% across all indications, ages, and treatment regimens, using data from four non-interventional post-authorization safety studies (PASS); this analysis was performed following changes in the preparation of raw material used to manufacture Octagam. Methods: All four studies included in- and out-patients prescribed Octagam for treatment of their medical condition. Physicians used case report forms to document baseline demographics, Octagam treatment details, and data on the efficacy of Octagam, and recorded all adverse drug reactions (ADRs) and other safety data. Results: Altogether 21,780 infusions of Octagam in 2,397 patients were included in our analysis. The most frequent indication for Octagam was secondary immunodeficiencies (SID; n = 1,368, 11,348 infusions), followed by primary immunodeficiencies (PID; n = 363; 3,923 infusions). During the individual patient observation, 83% of SID and 67% of PID patients were free of any infection. In up to 85% of all investigator assessments, Octagam was rated to have a favorable effect. In autoimmune diseases, investigators assessed Octagam as being beneficial in 70% (immune thrombocytopenia) up to 100% (Guillain-Barré syndrome), depending on the indication. The majority of patients (92%) tolerated Octagam treatment without any ADR. The overall incidence of reported ADRs was 1.0% for all infusions. The majority of ADRs were considered non-serious (93%) and mild or moderate (87%) in severity. No unexpected ADR signal was detected. Conclusions: This analysis demonstrates that the changes in the preparation of raw material used to manufacture Octagam did not affect the safety profile of Octagam^®^ 5% and 10%.

## Introduction 

Immunoglobulins are widely used in both, immunodeficient patients, to provide antibodies to prevent viral and bacterial infections (replacement therapy), and in patients with autoimmune and inflammatory diseases, mediating immunomodulation and attenuating inflammatory responses [1, 2, 3, 4, 5, 6, 7]. 

Octagam^®^ 5% (Octapharma AG, Lachen, Switzerland) is a sterile ready-to-use, sucrose-free liquid preparation of highly purified immunoglobulin for intravenous administration (IVIG), which was originally approved in Europe in 1995 for the treatment of primary (PID) [9] and secondary immunodeficiency (SID) as well as immune thrombocytopenia (ITP). It is now licensed in more than 80 countries worldwide. Octagam^®^ 10% was originally approved in Europe in 2008 for the same indications [8] and is now licensed in more than 50 countries. Both Octagam^®^ 5% and Octagam^®^ 10% are also approved as immunomodulation therapy in Guillain-Barré syndrome (GBS) and Kawasaki disease, chronic inflammatory demyelinating polyneuropathy (CIDP) in selected geographical areas and in allogenic bone marrow transplantation and congenital AIDS. 

In June 2011, Octagam^®^ 5% and Octagam^®^ 10% were available on the market with an amended manufacturing process. An additional chromatography step was introduced in the preparation of raw material paste used to manufacture Octagam to increase product safety. In order to test the positive risk-benefit ratio of the two Octagam products, a plan for integrated safety analysis was created with the objective to document and evaluate the tolerability and safety of Octagam^®^ 5% and 10% in any indication, age group, or treatment regimen. Data from two ongoing and two newly established non-interventional PASS studies was used to analyze the incidence and the nature of ADRs from at least 20,000 infusions with Octagam^®^ 5% or Octagam^®^ 10%. 

## Methods 

### Study design and conduct 

Data from four non-interventional, post-approval, multicenter studies were collected and included in this integrated safety analysis. The designs of these studies are presented in Table 1. 

For all four studies, in- and out-patients receiving Octagam^®^ 5% and Octagam^®^ 10% were included if their physicians had prescribed Octagam^®^ (5% or 10%) for their medical condition. Participating physicians decided the length and dosage of Octagam treatment based on disease type and severity as well as the patients’ clinical condition. 

### Data collection 

In all studies, physicians documented the data on case report forms (CRF) in accordance with each of the study protocols; for two studies, paper CRFs were used, while the other two studies used electronic CRFs (eCRFs). 

Collected information included baseline demographics (age, sex, weight, risk factors, indication, concomitant medications, and concomitant diseases) and Octagam treatment details (date and duration of infusions, dose, batch number, concomitant medication). Efficacy in preventing infections in patients with PID or SID was assessed by asking physicians to rate every 3 months the influence of treatment on patients’ disease as either “favorable”, “unchanged”, or “unfavorable” with regard to infection frequency, severity, duration, and antibiotic consumption. In patients with indications other than PID or SID, physicians were asked to rate in the 3-months observation periods how the patients’ underlying diseases had developed, using the outcome categories “improved”, “unchanged”, or “deteriorated”. One study also rated the influence of treatment on the course of the patients’ diseases at the end of the individual observation period as “beneficial”, “unchanged”, or “unfavorable”. 

In the course of the studies, physicians recorded all adverse drug reactions (ADRs) and other safety data. An ADR was defined as any noxious and unintended response to the observational drug, implying that a causal relationship between the observational drug and an adverse event is at least a reasonable possibility, i.e., a causal relationship cannot be ruled out. Adverse events that were not considered to be related to treatment with Octagam were not reported. ADRs were classified according to the nature of the event (MedDRA preferred term), frequency, severity (mild, moderate, severe), and causal relation to Octagam (definite, probable, possible, or unlikely). 

An independent Data Monitoring Committee (DMC) reviewed all safety relevant information, analyzed the nature of ADRs, and assessed the causality of serious ADRs in addition to the investigator and Octapharma. A definite relationship involved an adverse event that occurred within a reasonable time period from drug intake that made a causal relationship plausible, and that could not be explained by the disease or any other drug, and, for which re-challenge, if necessary, was satisfactory. An unlikely relationship involved an adverse event that occurred with a time from drug intake that made a causal relationship improbable but not impossible, and for which disease or other drugs could provide plausible explanations. In the case of unclear or implausible causality, the DMC made a final decision as to whether there was any likelihood of the adverse event being related to Octagam. 

The physicians were to report serious ADRs immediately (within 24 hours) to Octapharma. Serious ADRs were defined if at least one of the following criteria was applicable: event was life-threatening, required in-patient hospitalization or extended existing hospitalization, resulted in persistent or significant disability or incapacity, resulted in congenital anomaly/birth defect, or patient died. Non-serious ADRs as well as any other safety information were reported at the time of recognition if possible and no more than 10 working days thereafter. 

### Data processing 

Data from all four studies were entered into a common database. Data on paper CRFs were entered manually or transferred electronically into the database whereas data documented using eCRFs were transferred directly using a web-based electronic data-capture system. Where appropriate, data was stratified according to country and indication, age groups, or patient’s gender. 

### Statistical analysis 

Statistical analyses for this study were performed using the software package SAS release 9.3 (SAS Institute Inc., Cary, NC, USA). All safety and efficacy variables were analyzed by descriptive statistical analyses and included the absolute and relative frequencies, arithmetic mean, standard deviation (SD), minimum, median, and maximum, and two-sided 95% confidence intervals (CIs), where appropriate. 

### Ethics 

If required by the national regulations, the study protocol was reviewed and approved by each study site’s Independent Ethics Committee or Institutional Review Board before the start of the study. Patients provided written informed consent prior to study entry where required by local regulatory requirements. 

## Results 

### Patient and treatment characteristics 

In total, 2,397 patients, who had collectively received a total of 21,780 infusions of Octagam, were included in this safety analysis. Patients at any age with any indication for Octagam treatment were included (Table 2). The age ranged from 3 to 94 years with a mean of 60.4 years. The overall gender distribution was nearly equal with 46.6% males, but showed the typical unequal distribution in certain indications (e.g., 69% males among patients with CIDP and 82% females among multiple sclerosis (MS) patients). The most frequent indication for Octagam documented in this integrated safety analysis was SID (n = 1,368; 11,348 infusions), followed by PID (n = 363; 3,923 infusions), and ITP (n = 253; 1,599 infusions). For the majority of patients (59%), 5 or more infusions were documented within the project (Table 3) with a median treatment interval of 4.1 weeks. The mean dose of Octagam per treatment course differed between indications, with the lowest doses administered to patients with SID, MS, and PID (mean doses 0.2, 0.2, and 0.4 g/kg of bodyweight, respectively) and the highest doses administered to patients with dermatomyositis and pemphigus vulgaris (1.6 g/kg bodyweight each). More patients were treated with Octagam^®^ 5% (1,437 patients) than with Octagam^®^ 10% (961 patients) and some patients received both product strengths. 

### Tolerability 

**Overall tolerability**

**Figure 1.**
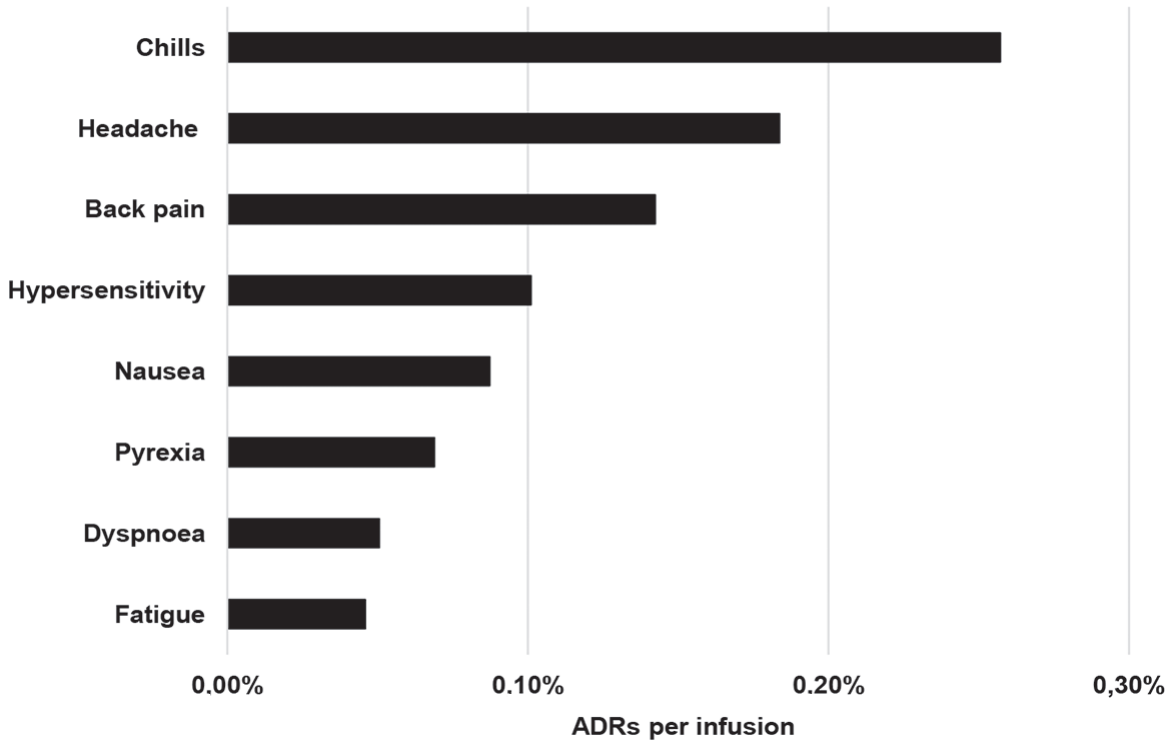
Frequency of adverse drug reactions (ADRs; % of all infusions), which were reported at least in 0.05% of infusions.

In total, 210 ADRs were reported in 191 patients during the observation period. The vast majority of ADRs were non-serious (93%) and mild or moderate (87%) in severity (Table 4). The overall incidence of reported ADRs per infusion was 1.0%. No trends with respect to the age or the sex of the patients and the incidences of ADRs were identified. The most commonly reported ADRs were chills, headache, back pain, and nausea (Figure 1). Of the 210 ADRs reported, 202 (96%) were recorded as having resolved and 5 (2%) as resolving; 2 outcomes were missing, and there was a single fatality. 

This integrated safety analysis showed that in most cases (88%) no premedication was used prior to infusion. Octagam was also well tolerated at high infusion rates. The maximum infusion rate was documented in two of the four studies. The documented maximum infusion rate for Octagam^®^ 5% was 8.7 mL/kg/h (14.5 mg/kg/min) and 7.2 mL/kg/h (12 mg/kg/min) for Octagam^®^ 10%. However, no correlation was found between infusion rates and ADR incidence. 

**Tolerability in patient subgroups**

Combined data from all four studies revealed some differences by indication in tolerability (Table 5). The most frequently experienced ADRs differed by indication, with headache being most frequent among patients with PID and chills the most frequent for patients with SID, whereas for ITP back pain was most frequent and for patients with MS, it was arthralgia. 

### Serious ADRs 

Out of 2,397 enrolled patients in four studies, 15 patients experienced at least 1 serious ADR (SADR). 10 SADRs occurred in SID patients, no SADRs were documented among PID and ITP patients. The remaining 5 SADRs occurred in patients with various other indications. 

The nature of these SADRs may be divided into 3 categories: The first category consists of 8 cases of immediate-type hypersensitivity reactions, which were assessed as related to Octagam by the investigators and by Octapharma. In 6 cases, the patients experienced chills and other symptoms either during the infusion or up to 4 hours after the infusion. Of these, 1 hypersensitivity case was assessed as mild and the patient received fluid replacement therapy; 3 hypersensitivity cases were assessed as moderate and were treated with fluid replacement in 1 case, glucocorticoids in the remaining cases and additional antibiotic therapy in 1 case; 2 cases were assessed as severe and were treated with glucocorticoids. The remaining 2 cases comprised severe headache during the infusion in 1 case and 1 circulatory insufficiency during infusion. However, all 8 patients recovered without sequelae. At least 2 patients continued the Octagam therapy after their event without further problems. 

The second category consists of 3 SADRs that can be classified as thromboembolic events (TEEs); 1 case each of thromboembolic occlusion of the aorta abdominalis, thromboembolic occlusion of the femoral artery, and pulmonary embolism. The total rate of TEEs per infusion across all four studies was 0.014% (3 per 21,780 infusions) The causal relationship to Octagam treatment was assessed as unlikely in 2 out of 3 cases and as possible in 1 case by the DMC. 

The third category of the remaining 4 SADRs comprises a heterogeneous group: Rash 28 days after last infusion, classified as unlikely related to Octagam treatment by Octapharma; renal colic 2 hours after last infusion, assessed as unlikely related to Octagam treatment by investigator and Octapharma; gastric ulcer 5 days after last infusion, assessed as unlikely related to Octagam treatment by investigator and Octapharma; the last case includes exacerbation of dyspnea during infusion, assessed as probably related to Octagam treatment by investigator. 

### Efficacy 

During the individual patient observation, 67% of PID and 83% of SID patients treated with Octagam were free of any infection. The most common infections concerned the upper respiratory tract in both groups. Physicians made 1,317 assessments for SID patients, with a mean assessment period of 3.4 months. The influence of Octagam on infection frequency was rated as “favorable” in 85% of patients. Furthermore, Octagam was rated to have favorable effects on infection severity, infection duration and the consumption of antibiotics in 83%, 79%, and 79% of patients, respectively. 

**Figure 2.**
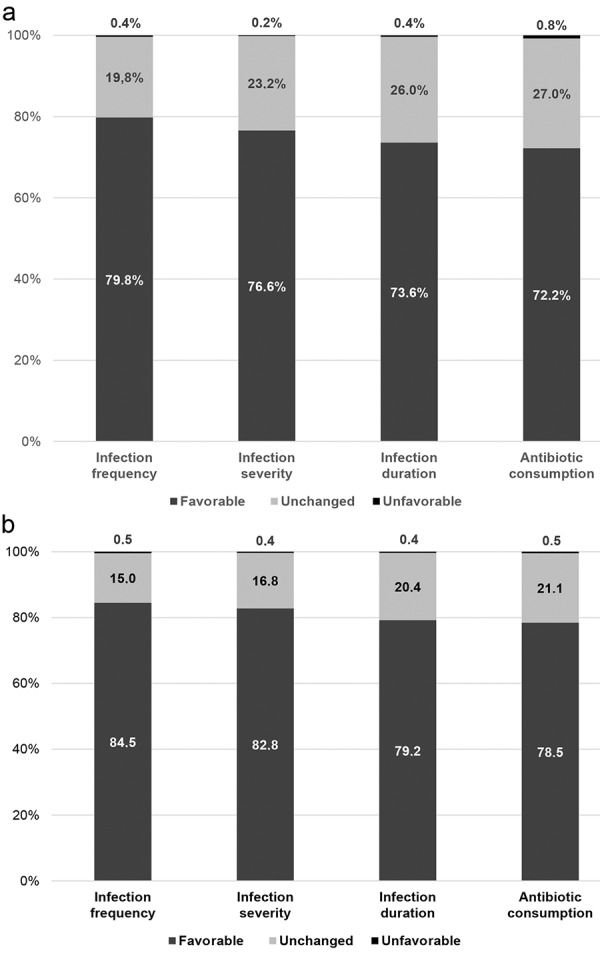
Proportion of patients with a) primary immunodeficiency (PID) and b) secondary immunodeficiency (SID) with favorable outcomes on Octagam treatment.

Among 530 assessments in PID patients, with a mean assessment period of 3.6 months, physicians considered “favorable” improvements in the frequency of infection in 80%, the severity of infection in 77%, the duration of infection in 74%, and the consumption of antibiotics in 72% (Figure 2); for all variables mentioned, a rating of “unfavorable” was given in only 0.2 – 0.8% of patients in both the PID and SID groups, with the remaining proportions rated as “unchanged”. 

For ITP patients, physicians made 125 assessments and rated the clinical appearance as “improved” in 70.4%. In only 0.8%, they rated the clinical appearance as “deteriorated” and the remainder (28.4%) was “unchanged”. In GBS, the influence of Octagam on the disease course was rated at the end of treatment: In all 4 cases, the rating was “beneficial”. In other neurologic autoimmune diseases, physicians made a total of 687 assessments, which revealed that the clinical appearance was stable in 72.3% or even improved in 26.1%. 

## Discussion 

Several review articles have addressed the safety of various IVIG preparations, finding that the most frequently reported adverse events following IVIG treatment are mild infusion-related reactions such as headache, nausea, fever, chills, and myalgia [10, 11, 12]. The risk of TEEs due to increased serum viscosity, particularly in patients with an already elevated serum viscosity, has also been noted with various IVIG preparations [11], as well as risks of renal tubular necrosis and aseptic meningitis [10], neither of the latter occurred in any patient in this study. 

Importantly, the adverse drug reaction profile observed in this study does not differ from that expected based on previous studies of Octagam and of other IVIG preparations. The ADRs reported in this analysis were similar to those observed in a 10-year prospective observational (non-interventional) study of Octagam in patients with various PID and SID and autoimmune diseases; these studies reported ADRs in 4.2% of all patients and in 0.35% of all infusions, with most ADRs graded as mild or moderate [13]. Findings regarding the most common types of ADRs were also similar to those of two previous prospective studies investigating the safety of Octagam [9, 14] Brenner et al. [14] reported on an open-label study in 54 patients treated with Octagam, in whom fever (4% of infusions) and chills (3%) were most frequent, followed by nausea and headache [14], with most adverse reactions graded as mild. In a more recent, open-label, 12-month study, conducted in 46 patients who received Octagam in a 3- or 4-week schedule chosen to best match their pre-existing infusion schedule, headache (3% of infusions) and nausea (0.6%) were the most common adverse events considered to be related to study medication, followed by chills, injection site reaction, back pain, and chest pain, with most adverse events mild to moderate in severity [9]. While the spectrum of common adverse events was generally similar, the incidence of ADRs reported in these two clinical trials was somewhat higher than in the present analysis of the four non-interventional PASS studies. In the study by Brenner et al. [14], ADRs were reported for 16% of all infusions, though relation to study medication was not reported. In the study published by Ochs et al. [9], adverse events that were suspected to be related to study medication and occurred within 30 minutes of an infusion were observed in 5.5% of infusions. In general, ADRs tend to be under-reported in routine medical care [15] and the rate of ADRs may be expected to be lower in a non-interventional study, in which patient data are gathered during routine treatments, than in a controlled clinical trial, in which diagnosis and treatment allocation follow a pre-specified protocol, and adverse events are rigorously documented. 

Results of the present analysis are consistent with a prospective audit conducted in 459 patients who were established on stable IVIG treatment having received at least six infusions uneventfully; as patients in this study were not selected, but rather the participating centers entered all patients receiving IVIG treatment, this study design is consistent with a non-interventional study. Over a total of 13,508 infusions given, 111 adverse reactions occurred, of which 91 were mild (headache, chills, nausea, itching) and 20 were moderate, with an overall adverse reaction rate of 0.8% [16]. Notably, this study did not find any differences between IVIG preparations. Furthermore, in a 2-year prospective, observational study in 117 patients with PID, with a total of 1,765 infusions (Octagam^®^ (Octapharma, Langenfeld, Germany), Tegeline^®^ (LFB-Biomedicaments, Les Ulis, France), Immunoglobulin^®^ (GCC, Suwon-City, Korea), Flebogama^®^ (Grifols, S.A., Barcelona, Spain), Vigam^®^ (Bio Products Laboratory Limited (BPL), Hertfordshire, UK), Kiovig^®^ (Baxter AG, Vienna, Austria), immediate infusion-related adverse reactions occurred in 38 of 1,765 infusions (2.15%), most commonly chills, fever, headache, nausea, malaise, and myalgia [17]. A recent observational study in 88 subjects with immunodeficiency or acute autoimmune diseases receiving IVIG 10% (Kiovig^®^) reported 67 ADRs in 27 subjects (30.6%), which were mild to moderate in intensity and mostly transient; the most frequent ADRs reported in this study were headache, pyrexia, vomiting, and chills [18]. In a multicenter, prospective, non-interventional study in 1,313 patients who were given a total of 21,995 Intratect^®^ (Biotest AG, Dreieich, Germany) infusions, there were 225 events attributed to Intratect^®^, resulting in an ADR rate of 1.0% per infusion [19]. 

While direct comparisons of the adverse event profiles of the various IVIG formulations are not possible, available data suggest that overall adverse event profiles are broadly similar. Any differences that do occur between formulations are likely explained by differences in manufacturing processes. Such variances may result in differences in the biochemical composition of the final product, including differences in sodium content, sugar content, osmolality, protein content, stabilizing agents and pH [20, 21, 22]. No hemolysis was observed during this project. 

From June 2011 on, an additional and now mandatory chromatography step to remove activated factor XI was integrated in the standard preparation of raw material used to manufacture Octagam. In addition, a Thrombin Generation Assay (TGA) was introduced in routine Octagam batch release (with the purpose to show that FXIa is below a limit defined by regulatory authorities). This safety analysis demonstrated that these new steps decreased the unexpected incidence of TEEs noticed in 2010 to rates observed in the original clinical studies of Octagam and improved the benefit-risk ratio of both Octagam preparations [8, 9]. An authority assessment of the Paul-Ehrlich-Institute in Germany confirmed that Octagam^®^ 5% and 10% are not associated with a high incidence of TEEs and this is consistent with the approximate frequency (incidence rate of 2 out of 10,000 infusions) (RMS Assessment Report Paul-Ehrlich-Institute; data on file). 

One of the strengths of this analysis is the fact that when data from multiple studies are pooled, the statistical power of the data can be increased. Additionally, the studies included in this analysis involved patients from several European countries as well as from the US. A further advantage is the inclusion of both Octagam product strengths and the use in various indications. 

There are some limitations to this safety analysis. First of all, there was a large variety in patient numbers in each of the studies analyzed. While this does not affect the primary analysis, any analysis by age or indication should be interpreted with caution. Furthermore, the fact of substantial variations between studies, due to the different study designs, various indications and different countries involved, must also be taken into account when interpreting the results of this study. 

## Conclusions 

This analysis demonstrated that the preparations of Octagam^®^ 5% and 10% investigated in this integrated safety analysis are well tolerated and safe. There were no unexpected safety issues and the ADR profile observed was consistent with that previously reported for Octagam and other IVIG products. These results demonstrate that the change in the manufacturing process has effectively re-established the positive risk-benefit ratio of the two Octagam products. In conclusion, Octagam was found to be safe and well tolerated over a broad group of patients and indications under conditions of routine medical use. 

## Acknowledgments 

The studies were funded by Octapharma. 

## Conflict of interest 

The authors were employees of Octapharma USA Inc., Hoboken, NJ, USA, Octapharma GmbH, Langenfeld, Germany or Octapharma Pharmazeutika Produktionsges.​m.b.H., Vienna, Austria at the time of study realization. 

**Table 1.** Design of the four non-interventional phase IV studies included in this safety analysis, using Octagam after changes in the preparation of the raw material used to manufacture it were implemented.

Study ID (Registration)	Study design	Country	Octagam strength	Study start and end dates	Period for integrated analysis (no. of patients)
Study 1 (ISRCTN58800347)	OL, MC, NIS, one-arm, non-controlled	Germany	10%	September 2008 – December 2013	June 2011 – November 2013 (803)
Study 2 (ISRCTN02245668)	OL, MC, NIS, one-arm, non-controlled	Austria, France, Spain, UK	5% and 10%	August 2011 – Ongoing	August 2011 – March 2014 (291)
Study 3 (NCT01859754)	OL, MC, two arms, controlled	USA	5%	May 2013 – Ongoing	May 2013 – March 2014 (83)
Study 4 (not registered)	OL, MC, NIS, one-arm, non-controlled	Germany	5%	February 1995 – December 2013	June 2011 – March 2013 (1,220)

MC = multicenter; NIS = non-interventional study; OL = open label.

**Table 2.** Baseline characteristics and demographics of patients included in the post-authorization safety analysis (n = 2,397).

Characteristic	
Patients, n	2,397
Gender, n (%)	
Male	1,117 (46.6)
Female	1,280 (53.4)
Mean age, years (range)	60.4 (3 – 94)
2 – 12 years, n (%)	8 (0.3)
13 – 17 years, n (%)	18 (0.8)
18 – 43 years, n (%)	372 (15.5)
44 – 68 years, n (%)	1,077 (44.9)
> 68 years, n (%)	922 (38.5)
Diagnosis, n	
PID	363
SID	1,368
ITP	253
Guillain-Barré syndrome	6
CIDP	58
MMN	17
Myasthenia gravis	16
Multiple sclerosis	163
Dermatomyositis	10
Polymyositis	12
Pemphigus vulgaris	3
Other	128

CIDP = chronic inflammatory demyelinating polyneuropathy; ITP = immune thrombocytopenia; MMN = multifocal motor neuropathy; PID = primary immunodeficiency; SID = secondary immunodeficiency.

**Table 3.** Overview of Octagam treatment.

Characteristic	
Infusions, n	21,780
Octagam^®^ 5%	12,222 (56.1%)
Octagam^®^ 10%	9,556 (43.9%)
Number of doses administered to each patient, n	
< 5	979 (41%)
≥ 5	1,409 (59%)
Median dose interval, weeks	4.1
Courses, n	19,126
Mean dose per infusion, g/kg of bodyweight	0.3
Mean dose per course, g/kg of bodyweight	0.4
Greatest infusion rate, mL/kg of bodyweight/hour	
Octagam^®^ 5%	8.7
Octagam^®^ 10%	7.2
Total Octagam weight used, kg	376.2

**Table 4.** Severity and seriousness of adverse drug reactions (ADRs; n = 210).

	Infusions with ADR	
	n	%
Severity		
Mild	96	46
Moderate	87	41
Severe	19	9
Missing	8	4
Seriousness		
Serious	15	7
Non-serious	195	93

**Table 5.** Most frequently reported ADRs per indication for the most common indications.

Indication group (total no. of infusions)	ADR	Total number of ADRs (n)	Incidence per infusion (%)
PID (3,923)	Headache	21	0.54
SID (11,348)	Chills	41	0.36
ITP (1,599)	Back pain	3	0.19

ITP = immune thrombocytopenia; PID = primary immunodeficiency; SID = secondary immunodeficiency.
